# Anisomycin activates JNK and sensitises DU 145 prostate carcinoma cells to Fas mediated apoptosis

**DOI:** 10.1038/sj.bjc.6600612

**Published:** 2002-11-04

**Authors:** J F Curtin, T G Cotter

**Affiliations:** Department of Biochemistry, University College Cork, Lee Maltings, Prospect Row, Cork, Ireland

**Keywords:** prostate, apoptosis, CD95, Fas, JNK

## Abstract

Treatment of the hormone refractory prostate cancer cell line DU 145 with sublethal concentrations of chemotherapeutic drugs has been reported to sensitise these cells to Fas mediated apoptosis. However, the mechanism by which this occurs has not been determined. Our group has shown that inhibition of JNK activity completely abrogates the effects of chemotherapeutic drugs. Using anisomycin, a potent JNK agonist, we have demonstrated a role for JNK in Fas mediated apoptosis in DU 145 cells. Inhibition of Caspase 8 and Caspase 9 completely inhibits this process which suggests that DU 145 cells require mitochondrial amplification of the Fas apoptotic signal. Furthermore, we have shown that inhibition of Fas mediated apoptosis is an early event in DU 145 cells, occurring upstream of Caspase 8 cleavage. It is hoped that identifying the target of JNK will allow novel therapies to be developed for the treatment of hormone refractory prostate cancer. Such therapies are especially important because no single or combined treatment to date has significantly prolonged survival in patients with hormone refractory prostate cancer.

*British Journal of Cancer* (2002) **87**, 1188–1194. doi:10.1038/sj.bjc.6600612 www.bjcancer.com

© 2002 Cancer Research UK

## 

Prostate cancer is the second most prevalent malignancy in the EU after lung cancer with about 200 000 new cases diagnosed and over 35 000 deaths each year. In England and Wales, 15 000 new cases and 8000 deaths are registered each year (Parkin *et al*, 2001). Although the prognosis is good for individuals with localised tumours, 10–20% of patients are diagnosed with metastatic prostate cancer (Crawford *et al*, 1999). These patients are usually treated with hormone ablation therapy which results in immediate tumour regression and temporary relief for the patient. However, hormone refractory prostate cancer invariably develops within 2–3 years of hormone ablation (Petrylak, 1999). This slowly proliferating cancer is extremely difficult to treat and the prognosis for the patient is generally poor. Over the past 5 years, chemotherapy has been used to improve the quality of life in patients with metastatic, hormone-refractory prostate cancer. No treatment has yet been found that cures the disease or even significantly prolongs survival (Petrylak, 1999).

Apoptosis, or programmed cell death (PCD), is characterised by morphological features including chromatin condensation, nuclear fragmentation, cell shrinkage, membrane blebbing and apoptotic body formation (Kerr *et al*, 1972). Although a variety of different environmental insults and signalling pathways can stimulate apoptosis in cells, most of these signals converge at a family of cysteine proteases called the caspases. Like many proteases, they are synthesised in an inactive form and cleavage into active caspases is essential for the proliferation of the apoptotic signal. Caspases can be divided into two main subfamilies, initiator caspases and effector caspases (Wolf and Green, 1999). The Fas receptor is a member of the Tumour Necrosis Factor receptor superfamily and is expressed at the plasma membrane in a variety of tissues. Ligation of Fas ligand or a cross-linking antibody to the Fas receptor induces apoptosis in susceptible cells. Fas receptor clustering results in the recruitment and auto-cleavage of the initiator caspase, Procaspase 8, at the plasma membrane. Active Caspase 8 proceeds to cleave downstream cellular targets including the effector Caspases 3 and 7, and the Bcl-2 family member Bid (Peter and Krammer, 1998). Often an amplification step is required for Caspase 3 cleavage and morphological apoptosis. Caspase 8 cleaves Bid into tBid, a pro-apoptotic Bcl-2 family member that induces cytochrome *c* release and apoptosome formation. This amplification loop through the mitochondrion drives the apoptotic programme in type II cells (Scaffidi *et al*, 1999b).

DU 145 cells, a hormone refractory prostate adenocarcinoma, are highly resistant to Fas mediated apoptosis *in vitro*. In a study performed using cell lines derived from prostate tumours with different pathological stages including DU 145, it was observed that ALVA-31 and PPC-1 were sensitive to Fas mediated apoptosis. These were reported to be isolated from primary prostatic tumours. In contrast, the cell lines LNCaP, DU 145 and PC-3 were resistant. These cell lines were reported to be derived from distant metastases. The authors correlated prostate cancer disease progression with resistance to Fas. Furthermore they suggest that this phenomenon may explain, at least in part, the inability to treat hormone refractory prostate cancer (Hedlund *et al*, 1998). The two other cell lines used in this study, JCA-1 and TSU-Pr1 have since been reclassified as bladder cancer cell lines (van Bokhoven *et al*, 2001).

In order to study the resistance of hormone refractory prostate cancer to chemotherapy, the effects of chemotherapeutic drugs on DU 145 cells was explored (Uslu *et al*, 1997; Costa-Pereira and Cotter, 1999). Our group discovered that sublethal concentrations of camptothecin, a novel topoisomerase I inhibitor, sensitised DU 145 cells to Fas mediated apoptosis by 20-fold (Costa-Pereira and Cotter, 1999). Activation of the stress kinase JNK was found to be essential in potentiating Fas mediated apoptosis (Costa-Pereira *et al*, 2000). In this study, we use anisomycin, a potent activator of JNK, to underscore the role played by JNK in Fas mediated apoptosis in DU 145 cells.

## MATERIALS AND METHODS

### Cell lines and reagents

DU 145 and Jurkat T cells were obtained from American Type Culture Collection (ATCC, Rockville, MD, USA). Cell culture reagents were purchased from Gibco BRL (UK) with the exception of foetal calf serum (FCS) (Sigma, UK). Anisomycin was purchased from Sigma (UK) and was dissolved in DMSO at a concentration of 5 mg ml^−1^. A working stock at 10 μg ml^−1^ in RPMI was prepared from the original stock. A FACScan (Beckton Dickinson, BD Biosciences, Germany) and Cell Quest software Version 3.3 (Beckton Dickinson) were used for all flow cytometry assays. Annexin V-FITC was purchased from Bender MedSystems (Germany) and propidium iodide (PI) from Sigma (UK). TUNEL reagents were obtained from Roche (UK) and JC-1 was purchased from Molecular Probes (Netherlands). The antibodies used in this study were mouse anti-Fas IgM clone CH11, mouse anti-Fas IgG for flow cytometry (Bender MedSystems, Germany), phospho-JNK (Thr183/Tyr185) clone G9 and rabbit anti-Caspase 3 (Cell Signalling Technology, New England Biolabs UK), rabbit anti-JNK1 and rabbit anti-Fas ligand (Santa Cruz, USA), rabbit anti-Bid (BioSource International, USA), rabbit anti-human caspase 8 (R&D Systems, UK) and mouse anti-β Actin clone AC-15 (Sigma, UK). All FITC and R-Phycoerythrin conjugated secondary antibodies were purchased from Sigma (UK) and HRP conjugated secondary antibodies from DAKO (Denmark). The Caspase inhibitors z-IETD-fmk and z-LEHD-fmk were obtained from Calbiochem (CN Biosciences UK). Enhanced Chemiluminescence Reagent (ECL) was purchased from Amersham Biosciences (UK).

### Cell culture and treatment

DU 145 cells were cultured in RPMI 1640 medium supplemented with 5% FCS, 2 mM L-Glutamine in the presence of 10 IU ml^−1^ penicillin-streptomycin. Jurkat cells were cultured in RPMI 1640 medium containing 10% FCS, 2 mM L-Glutamine and 10 IU ml^−1^ penicillin-streptomycin. Cells were cultured at 37°C in a humidified atmosphere with 5% CO_2_ and were routinely subcultured every 2–3 days. Prior to every experiment DU 145 cells were grown to 75% confluency and Jurkats were resuspended at 0.5 million ml^−1^. DU 145 cells were pretreated with 250 ng ml^−1^ anisomycin for 10 min before addition of 200 ng ml^−1^ anti-Fas IgM.

### Annexin V binding and propidium iodide uptake assay

DU 145 and Jurkat cells were incubated with 250 ng ml^−1^ anisomycin and 200 ng ml^−1^ anti-Fas IgM for 8 h. The cells were subsequently harvested, washed once in PBS and resuspended in Annexin V binding buffer (150 mM NaCl, 18 mM CaCl_2_, 10 mM HEPES, 5 mM KCl, 1 mM MgCl_2_). FITC conjugated Annexin V (1 μg ml^−1^), which binds specifically to apoptotic cells, was added to each sample and incubated at ambient temperature for 5 min. Propidium iodide (50 μg ml^−1^), excluded from viable cells, was added immediately prior to reading the samples on the FACScan. Where indicated the Caspase 8 and Caspase 9 inhibitors were incubated for 10 min at 50 μM prior to treating the cells with anisomycin or Fas.

### DNA fragmentation assay

TUNEL measures DNA fragmentation using the enzyme Terminal deoxynucleotide Transferase (TdT) to transfer multiple biotin labelled nucleotides to the 3′ hydroxyl groups of DNA. FITC conjugated Avidin can be used to stain this modified label. Using flow cytometry, cells with fragmented DNA in their nuclei display an increased fluorescent signal in the FL-1 channel relative to untreated cells.

DU 145 and Jurkat cells were harvested, washed twice in PBS and fixed slowly in 1% paraformaldehyde (PFA) on ice for 15 min. The cells were washed twice in PBS and resuspended in 25 μl reaction mixture (TdT buffer, 2.5 mM CoCl_2_, Bio-16-dUTP and TdT enzyme). The DNA labelling reaction was allowed to proceed for 30 min at 37°C. The cells were washed twice in PBS and resuspended in 50 μl staining buffer (5×SSC, 5% w v^−1^ dry milk, 1×Avidin-FITC and 1/1000×Triton X-100). The cells were stained for 30 min at room temperature in the dark and washed twice in PBS. Flow cytometry and Cell Quest were used to collect and analyse the data.

### Fas receptor expression

0.5×10^6^ cells were harvested per sample and washed twice in PBS. They were stained for 1 h at 4°C with 20 μg ml^−1^ of the primary antibody mouse anti-Fas IgG. After another two washes with PBS, the cells were stained with the FITC conjugated secondary antibody sheep anti-mouse IgG for 1 h at 4°C in the dark. Cells stained with secondary antibody alone were used to compensate for intrinsic fluorescence and non-specific binding of the secondary antibody. The cells were washed twice in PBS and the presence of Fas R was detected in FL-1 using a FACScan flow cytometer.

### Fas ligand expression

0.5×10^6^ cells were harvested per sample and were fixed slowly in ice cold 1% PFA for 15 min. The cells were permeabilised overnight in 70% ethanol (−20°C) and stained with 2 μg ml^−1^ rabbit anti-Fas ligand or 2 μg ml^−1^ rabbit irrelevant IgG in IFA_TX_ (4% FCS, 150 mM NaCl, 10 mM HEPES, 0.1% sodium azide, 0.1% Triton X-100) for 1 h at 4°C. Subsequently, the cells were stained for 1 h with 12 μg ml^−1^ FITC-conjugated anti-rabbit IgG in the dark at 4°C. Fas ligand expression was analysed on the FACScan using Cell Quest software.

### Mitochondrial membrane depolarisation

The lipophilic cation called JC-1 is cell permeable and selectively accumulates in the mitochondria of live cells. When depolarisation of the mitochondria occurs, the emission spectrum of JC-1 changes from 590 nm (its aggregated form) to 530 nm (its monomeric form) and this can be analysed using flow cytometry. Depolarisation of mitochondria results in an increase in fluorescence in the FL-1 channel, and a concurrent decrease in the FL-2 channel in flow cytometers.

DU 145 cells were harvested, resuspended in RPMI+10% FCS and 2.5 μg ml^−1^ JC-1 was added. The samples were incubated at room temperature for 20 min in the dark, washed twice in PBS and read on the FACScan. Analysis was carried out using Cell Quest software.

### SDS–PAGE and Western blot

Protein extracts were prepared from cells using RIPA lysis buffer (50 mM Tris, pH 7.4; 150 mM NaCl; 1 mM each of NaF, NaVO_4_ and EGTA; 1% NP40; 0.25% sodium deoxycholate; 0.2 mM phenylmethylsulphonyl fluoride; 1 μg ml^−1^ each of antipain, aprotinin and chymostatin; 0.1 μg ml^−1^ leupeptin; 4.0 μg ml^−1^ pepstatin) and 30 μg of protein was loaded in each lane of an SDS polyacrylamide gel. Electrophoresis and Western blotting was subsequently carried out. Non-specific protein binding sites on the membrane were blocked using 5% dry milk in TBS+0.1% Tween-20 for 1 h at room temperature. The membrane was stained with primary and peroxidase conjugated secondary antibodies according to the manufacturer's recommended protocol and labelled protein was detected using ECL.

## RESULTS

### Anisomycin activates JNK and sensitises DU 145 cells to Fas mediated apoptosis

**Figure 1:**
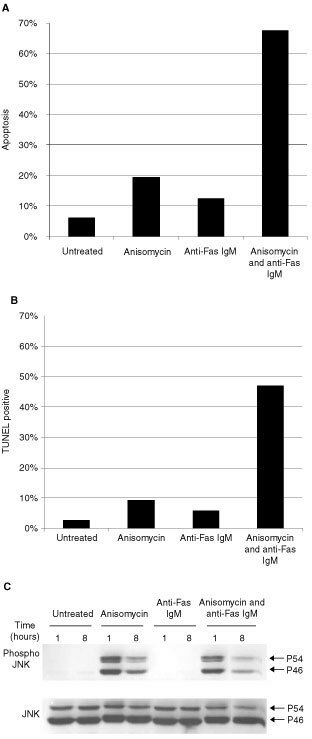
Anisomycin sensitised the androgen independent cell line DU 145 to Fas mediated apoptosis. DU 145 cells were pre-treated with anisomycin (250 ng ml^−1^) for 10 min before the addition of anti-Fas IgM (200 ng ml^−1^). Cells were stained with Annexin V and PI (**A**) or TUNEL (**B**) after 8 h incubation as described in the Materials and Methods section. Flow cytometry was used to visualise the extent of apoptosis. Data are representative of at least three independent experiments. (**C**) Anisomycin, not anti-Fas IgM, stimulates prolonged JNK activation in DU 145 cells. Western blot analysis was used to detect active JNK in untreated DU 145 cells, or cells treated with anisomycin (250 ng ml^−1^), anti-Fas IgM (200 ng ml^−1^) or both anisomycin and anti-Fas IgM for 1 and 8 h. Total JNK expression was determined to ensure equal protein loading.

The hormone refractory cell line DU 145 is highly resistant to Fas mediated apoptosis. This appears to be a common event during prostate cancer progression. Cell lines isolated from early stages of prostate cancer are usually sensitive to the Fas activating antibody anti-Fas IgM. Those cell lines derived from secondary tumours after hormone ablation therapy are generally resistant to Fas (Hedlund *et al*, 1998). Our group has previously shown that camptothecin, a Topoisomerase I inhibitor, sensitises DU 145 cells to Fas mediated apoptosis (Costa-Pereira and Cotter, 1999). Additional analysis identified activation of the stress kinase JNK as an integral event in this process (Costa-Pereira *et al*, 2000). In order to understand the mechanisms behind this sensitisation process we have used anisomycin, an agonist of the JNK pathway in mammalian cells, which is often used in studies involving JNK because of its specificity and potency in activating the JNK pathway. As expected, we found that anisomycin can act in synergy with Fas to induce apoptosis in DU 145 cells. Phosphatidylserine flipping, an early event during apoptosis was detected with FITC-conjugated Annexin-V using flow cytometry. Propidium iodide was used as a counter stain to distinguish between early and late apoptosis (Figure 1A

). Flow cytometry was also used to detect DNA fragmentation, another hallmark of apoptosis, in TUNEL labelled DU 145 cells following incubation with anisomycin (250 ng ml^−1^) and anti-Fas IgM (200 ng ml^−1^) (Figure 1B). This rapid onset of DNA fragmentation in our system is indicative of a stronger apoptotic stimulus when anisomycin is used to sensitise DU 145 cells to anti-Fas IgM in comparison with other cytotoxic drugs. Extensive DNA fragmentation was only observed after 48 h when CPDD and CHX were used (Rokhlin *et al*, 1997; Uslu *et al*, 1997). Numerous reports have been described in the literature of both Caspase 8 dependent and Caspase 8 independent JNK activation during Fas mediated apoptosis. We used an antibody specific to phosphorylated JNK to assess the status of JNK activation in DU 145 cells after 1 and 8 h incubation with anisomycin (250 ng ml^−1^) and anti-Fas IgM (200 ng ml^−1^). We verified that JNK is not activated either transiently (1 h) or prolonged (8 h) with anti-Fas IgM (Figure 1c). As expected, anisomycin was found to stimulate prolonged JNK activation in DU 145 cells. An *in vitro* kinase assay using radiolabelled ^32^P was used to verify the activity of JNK (data not shown).

### Fas receptor and Fas ligand are not up-regulated by anisomycin in DU 145 cells

**Figure 2:**
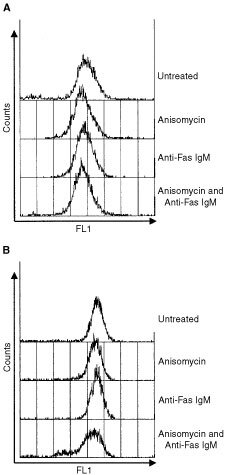
Flow cytometric analysis of Fas receptor (**A**) or Fas ligand (**B**) expression in untreated DU 145 cells or following 8 h incubation with anisomycin (250 ng ml^−1^), anti-Fas IgM (200 ng ml^−1^) or both. Data are representative of at least three independent experiments and similar data were obtained for incubations of 1, 2 and 4 h.

Various reports have shown that down-regulation of Fas receptor or Fas ligand expression occurs in some cancer cells. In addition, expression of Fas ligand has been reported to increase following JNK activation in Jurkat cells (Herr *et al*, 2000). This increase in Fas ligand expression caused an increase in the kinetics of Fas mediated apoptosis. We assessed the expression of both Fas receptor and Fas ligand over an 8 h period (1,2,4 and 8 h) following incubation with anisomycin (250 ng ml^−1^) or anti-Fas IgM (250 ng ml^−1^). Cell surface Fas receptor was expressed on 95% of DU 145 cells and its expression was not found to change following treatment with anisomycin or anti-Fas IgM (Figure 2A

). Similarly, Fas ligand was expressed in 90% of DU 145 cells and expression was not increased in DU 145 cells following incubation with anisomycin or anti-Fas IgM (Figure 2B). Western blot analysis confirmed that the expression of Fas receptor and Fas ligand was not upregulated following drug treatment (data not shown).

### Activation of Caspase 3 during Fas mediated apoptosis

**Figure 3:**
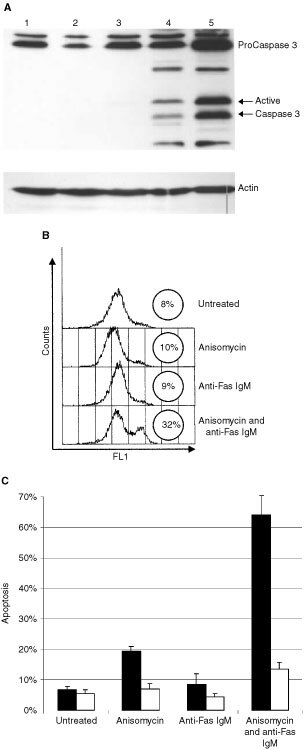
(**A**) Untreated DU 145 (**1**) cells or cells treated for 8 h with anisomycin (250 ng ml^−1^) (**2**), anti-Fas IgM (200 ng ml^−1^) (**3**) or both (**4**) were stained for Caspase 3 cleavage. Jurkats treated with anti-Fas IgM (200 ng ml^−1^) for 4 h were used as a positive control (**5**). (**B**) Mitochondrial membrane depolarisation (ΔΨ) was detected in DU 145 cells using the lipophilic JC-1 probe, as described previously. ΔΨ was only observed in DU 145 cells treated for 8 h with both anisomycin (250 ng ml^−1^) and anti-Fas IgM (200 ng ml^−1^). (**C**) DU 145 cells were pre-treated with 50 μM z-LEHD-fmk (white columns) or a DMSO control (black columns) for 10 min before treating with anisomycin (250 ng ml^−1^) and anti-Fas IgM (200 ng ml^−1^) for 8 h. Apoptosis was assessed by staining the cells with Annexin V-FITC and PI. The error bars represent standard deviation after three independent experiments.

In order to delineate the mechanisms by which DU 145 cells are sensitised to Fas mediated apoptosis we analysed the major events during Fas mediated apoptosis. Most apoptotic stimuli converge on Caspase 3, a cysteine protease and the main effecter caspase during Fas mediated apoptosis. Once activated, Caspase 3 cleaves a variety of substrates responsible for the morphological and biochemical changes observed during apoptosis (Nicholson, 1999). We found that both anisomycin and anti-Fas IgM treatment alone were insufficient for Caspase 3 activation in DU 145 cells. However, co-incubation of cells with both anisomycin and anti-Fas IgM clearly potentiates the activation of Caspase 3 in DU 145 cells (Figure 3A

). Anti-Fas IgM treated Jurkats are used as a positive control for Caspase 3 activation. Flow cytometric analysis confirmed that 70% of DU 145 cells expressed the active form of Caspase 3 following treatment with both anisomycin and anti-Fas IgM (data not shown).

### Mitochondrial membrane depolarisation

It has been shown that Fas receptor activation is often not sufficient for direct activation of Caspase 3. These cells, known as type II cells, require an amplification signal through the mitochondrion. Caspase 8 cleaves and activates Bid, a pro-apoptotic Bcl-2 family member. This results in mitochondrial membrane depolarisation, cytochrome *c* release and amplification of the Fas apoptotic signal through Caspase 9 (Kim *et al*, 2000). Here we used the voltage sensitive, lipophilic fluorescent probe JC-1 to analyse the extent of mitochondrial membrane depolarisation in DU 145 cells. Depolarisation of the mitochondrion causes an increase in FL-1 fluorescence and a concomitant decrease in FL-2 fluorescence when analysed by flow cytometry (Cossarizza *et al*, 1993). We found that stimulation of DU 145 cells for 8 h with anisomycin (250 ng ml^−1^) or anti-Fas IgM (200 ng ml^−1^) alone did not result in permeability transition of the mitochondria (Figure 3B). This suggested that Fas mediated apoptosis is inhibited up-stream of mitochondrial depolarisation in DU 145 cells. Incubation of DU 145 cells with both anisomycin (250 ng ml^−1^) and anti-Fas IgM (200 ng ml^−1^) resulted in mitochondrial depolarisation. We found that incubation of DU 145 cells with 50 μM z-LEHD-fmk (a Caspase 9 specific inhibitor) completely abrogated apoptosis when incubated with anisomycin (250 ng ml^−1^) and anti-Fas IgM (200 ng ml^−1^) (Figure 3C). This suggested that mitochondrial membrane depolarisation and cytochrome *c* release are essential events for Fas mediated apoptosis in DU 145 cells.

### Caspase 8 activation in DU 145 cells

**Figure 4:**
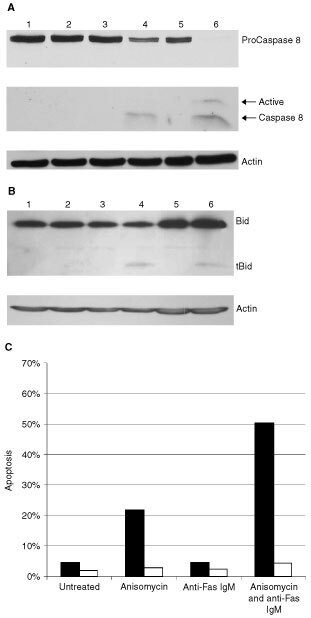
Inhibition of Fas mediated apoptosis occurs upstream of Caspase 8 cleavage in DU 145 cells. (**A**) Western blot analysis of Caspase 8 in untreated DU 145 cells (**1**) or following incubation with anisomycin (250 ng ml^−1^) (**2**), anti-Fas IgM (200 ng ml^−1^) (**3**) or both (**4**) for 8 h. Untreated (**5**) and anti-Fas IgM treated (200 ng ml^−1^ anti-Fas IgM, 4 h) (**6**) Jurkat cells were used as a positive control for the P14 and P10 Caspase 8 cleavage products. β-Actin was also probed to ensure equal protein loading. (**B**) Bid expression and cleavage was analysed by Western blot in untreated DU 145 cells (**1**) or in cells incubated with anisomycin (250 ng ml^−1^) (**2**), anti-Fas IgM (200 ng ml^−1^) (**3**) or both (**4**) for 8 h. Jurkats untreated (**5**) or treated with anti-Fas IgM (200 ng ml^−1^) for 4 h (**6**) are used as a positive control. (**C**) DU 145 cells were pre-treated with 50 μM z-IETD-fmk (white columns) or a DMSO control (black columns) for 10 min before treating with anisomycin and anti-Fas IgM as before. Apoptosis was determined by staining with both Annexin V and PI after 8 h. Data is representative of three independent experiments.

The proximal caspase in the Fas apoptotic pathway is Caspase 8. Recruitment and auto-cleavage of Procaspase 8 occurs following Fas receptor activation in sensitive cells. Active Caspase 8 is tetrameric, consisting of two P14 and two P10 subunits. Using Western blot analysis, we found that Caspase 8 is not cleaved in DU 145 cells following treatment with anti-Fas IgM (200 ng ml^−1^) (Figure 4A

). Therefore, we concluded that inhibition of Fas mediated apoptosis occurred upstream of Caspase 8 activation in DU 145 cells. Caspase 8 cleavage products were only evident following incubation with both anisomycin (250 ng ml^−1^) and anti-Fas IgM (200 ng ml^−1^). This was also true for the Caspase 8 substrate Bid (Figure 4B). Z-IETD-fmk (50 μM), an irreversible inhibitor specific to Caspase 8, was found to completely protect against apoptosis induced by anisomycin (250 ng ml^−1^) and anti-Fas IgM (200 ng ml^−1^) (Figure 4C). Interestingly, this inhibitor also abolishes apoptosis associated with anisomycin alone. This suggests that low levels of Caspase 8 activation occur in DU 145 cells following incubation with anisomycin alone. A dose titration of z-IETD-fmk confirmed that this inhibitor specifically inhibits Caspase 8 at 50 μM (data not shown). FLIP is a family of proteins structurally related to Caspase 8 that inhibit Fas mediated apoptosis when overexpressed in cells (Scaffidi *et al*, 1999a). Two main isoforms of FLIP are expressed in cells, a long splice variant (FLIP_L_) and a short splice variant (FLIP_S_). Using Western blot analysis we found that DU 145 cells express FLIP_S_. However, expression of this caspase 8 inhibitor does not appear to decrease following incubation with anisomycin (data not shown).

## DISCUSSION

The sensitivity of prostate cancer cell lines to Fas mediated apoptosis has been shown to correlate with tumour stage, grade and resistance to chemotherapeutic drugs (Hedlund *et al*, 1998). Our group and others have shown that DU 145 cells are highly resistant to Fas mediated apoptosis. Co-treatment with sublethal concentrations of chemotherapeutic drugs including cyclohexamide (CHX), cisplatin (CPDD), etoposide (VP16) and camptothecin was found to sensitise these cells to Fas mediated apoptosis (Uslu *et al*, 1997; Rokhlin *et al*, 1998; Costa-Pereira and Cotter, 1999) independently of new protein synthesis (Frost *et al*, 1999). Our group subsequently identified a key role for JNK in this process. DU 145 cells were co-treated with camptothecin and anti-Fas IgM and were completely protected from apoptosis by anti-sense oligonucleotides specific for JNK (Costa-Pereira *et al*, 2000). In addition, camptothecin is a potent activator of JNK and sensitises DU 145 cells to Fas mediated apoptosis to a much greater extent than CHX, CPDD and VP16 (Costa-Pereira and Cotter, 1999). We have shown that anisomycin, a potent activator of JNK in mammalian cells, sensitises DU 145 cells to Fas mediated apoptosis to a similar extent as camptothecin. We felt that because camptothecin is also a topoisomerase I inhibitor and the mechanisms by which it activates JNK are unclear, anisomycin would present a better option for delineating the effects of JNK during Fas induced apoptosis.

Binding of Fas ligand, or Fas activating antibodies, to Fas receptor results in DISC formation and prolonged JNK activation by either Caspase 8 dependent or Caspase 8 independent mechanisms (Chang *et al*, 1998; Rudel *et al*, 1998; Charette *et al*, 2001; Graves *et al*, 2001). We have shown that stimulating Fas R with anti-Fas IgM alone does not result in JNK activation in DU 145 cells. We found that mitochondrial membrane depolarisation only occurs in DU 145 cells co-stimulated with anisomycin and anti-Fas IgM. In addition Caspase 8 and Bid were only cleaved following incubation with both anisomycin and anti-Fas IgM. This suggests that anisomycin sensitises DU 145 cells to Fas mediated apoptosis at a point upstream of Caspase 8 cleavage, probably during DISC formation.

There are some reports of caspase independent cell death following Fas R activation. These are mediated through kinases such as RIP and ASK1 (Holler *et al*, 2000; Charette *et al*, 2001). However, we have shown that both Caspase 8 and Caspase 9 inhibitors completely abrogate apoptosis induced by anisomycin and anti-Fas IgM in DU 145 cells. Therefore, anisomycin sensitised DU 145 cells to apoptosis mediated by Fas that is dependent on both Caspase 8 activity to initiate the pathway and Caspase 9 activity as an amplification step required for Caspase 3 activation and apoptosis.

The principal apoptotic pathway activated by many anti-cancer drugs is the Fas apoptotic pathway. DU 145 cells incubated with toxic concentrations of 9-amino camptothecin were found to increase Fas receptor and Fas ligand expression and decrease c-FLIP_S_. Apoptosis could be inhibited by transient overexpression of c-FLIP_S_, suggesting that 9-amino camptothecin induces apoptosis through the Fas apoptotic pathway (Chatterjee *et al*, 2001). However, we found no evidence for increased Fas receptor or Fas ligand expression following incubation with anisomycin. We did observe a decrease in Fas ligand expression after apoptotic body formation in cells incubated with both anisomycin and anti-Fas IgM. This is most probably due to the shedding of membrane bound Fas ligand and is irrelevant to the initiation of apoptosis in these cells. We also analysed FLIP_S_ expression but no changes were observed following treatment with anisomycin. Chemotherapeutic drugs have also been reported to activate the Fas apoptotic pathway without upregulation of Fas ligand or Fas receptor. Apoptosis induced by CPDD, VP16 and vinblastine (VB) was shown involve Fas receptor clustering and Caspase 8 activation and was independent of Fas ligand in various colon cancer cells and leukaemia cells (Micheau *et al*, 1999). It is possible that anisomycin is inducing Fas receptor aggregation independently of anti-Fas IgM but this is highly unlikely in light of our results. It seems more probable that JNK activation enhances Fas receptor aggregation and DISC formation through its interaction with some key regulator of DISC formation in DU 145 cells.

Conventional chemotherapy has been unsuccessful in treating prostate cancer. No single or combined chemotherapy regime has been identified that significantly enhances long term survival. This may be due, at least in part, to the resistance developed to Fas mediated apoptosis in hormone refractory prostate cancer. We have sensitised DU 145 cells to Fas mediated apoptosis using the JNK agonist anisomycin. In addition we have traced the effects of JNK to a point upstream of Caspase 8 cleavage. It is hoped that by understanding this process novel drug targets may be identified that improve the treatment of hormone refractory prostate cancer.
